# Evidence for extensive heterotrophic metabolism, antioxidant action, and associated regulatory events during winter hardening in Sitka spruce

**DOI:** 10.1186/1471-2229-13-72

**Published:** 2013-04-30

**Authors:** Eva Collakova, Curtis Klumas, Haktan Suren, Elijah Myers, Lenwood S Heath, Jason A Holliday, Ruth Grene

**Affiliations:** 1 Department of Plant Pathology, Physiology, and Weed Science, Virginia Tech, Blacksburg, VA, 24061, USA; 2 Genetics, Bioinformatics and Computational Biology Program, Virginia Tech, Blacksburg, VA, 24061, USA; 3 Department of Forest Resources and Environmental Conservation, Virginia Tech, Blacksburg, VA, 24061, USA; 4 Department of Computer Science, Virginia Tech, Blacksburg, VA, 24061, USA

**Keywords:** Microarray, Sitka spruce, Carbon metabolism, Cell walls, Adaptation mechanisms, Visualization

## Abstract

**Background:**

Cold acclimation in woody perennials is a metabolically intensive process, but coincides with environmental conditions that are not conducive to the generation of energy through photosynthesis. While the negative effects of low temperatures on the photosynthetic apparatus during winter have been well studied, less is known about how this is reflected at the level of gene and metabolite expression, nor how the plant generates primary metabolites needed for adaptive processes during autumn.

**Results:**

The MapMan tool revealed enrichment of the expression of genes related to mitochondrial function, antioxidant and associated regulatory activity, while changes in metabolite levels over the time course were consistent with the gene expression patterns observed. Genes related to thylakoid function were down-regulated as expected, with the exception of plastid targeted specific antioxidant gene products such as thylakoid-bound ascorbate peroxidase, components of the reactive oxygen species scavenging cycle, and the plastid terminal oxidase. In contrast, the conventional and alternative mitochondrial electron transport chains, the tricarboxylic acid cycle, and redox-associated proteins providing reactive oxygen species scavenging generated by electron transport chains functioning at low temperatures were all active.

**Conclusions:**

A regulatory mechanism linking thylakoid-bound ascorbate peroxidase action with “chloroplast dormancy” is proposed. Most importantly, the energy and substrates required for the substantial metabolic remodeling that is a hallmark of freezing acclimation could be provided by heterotrophic metabolism.

## Background

The transition from active growth to dormancy in woody perennials of the temperate and boreal regions involves molecular, cellular, and whole-plant responses. Triggered by gradual decreases in temperature and day length, this remodeling reflects a complex array of adaptations to the physical and metabolic stress imposed by freezing temperatures. Substantial changes in the transcriptome and metabolome accompany this transition and enable the plant to avoid cellular damage resulting from direct thermal effects of freezing temperatures on macromolecules, extracellular ice formation, and the generation of reactive oxygen species (ROS). In deciduous angiosperms and evergreen gymnosperms, the light- and hormone-regulated transcriptional and metabolic changes associated with cambial and vegetative bud dormancy and freezing tolerance are initiated by prolonged nights long prior to the actual freezing [1]–[7].

Because most conifers retain their leaves (needles) during winter, protection from freezing has to extend to these photosynthetic organs. The photosynthetic apparatus is prone to oxidative damage as temperatures drop due to energetic and metabolic imbalance [8]. This imbalance is a result of the temperature dependency of metabolic processes, including photochemistry. Light is absorbed by photosystems regardless of the temperature, but enzymes are inhibited at low temperatures, resulting in over-reduction of the photosystems and photoinhibition. High-energy electrons produced in excess reduce molecular oxygen to generate ROS causing photosystem damage [9]. In evergreen conifers, such oxygen reduction in a Mehler-type reaction is coupled to antioxidant defenses and represents an important mechanism of dissipating excess energy absorbed by photosystem I at low temperatures [8,10].

Photosynthetic acclimation to low temperatures and freezing in evergreen gymnosperms also involves other mechanisms, including the re-localization of chloroplasts, reorganization and aggregation of photosystem antennae, chlororespiration, and non-photochemical quenching leading to conversion of absorbed light energy to heat rather than to reductant for CO_2_ fixation and growth [8,11]–[14]. Lodgepole pine (*Pinus contorta*) was shown to reduce the antenna size and the number of reaction centers in photosystem II during winter hardening to minimize light absorption [15]. Additional mechanisms that facilitate the transition from energy harvesting to energy dissipation that occurs with winter hardening include changes in abundance of specific thylakoid proteins [16], non-photochemical quenching via the xanthophyll cycle, and antenna protonation via thylakoid pH changes to dissipate the excess energy to heat [8]. A second, zeaxanthin-independent, quenching mechanism has also been described, involving charge recombination between photosystem II reaction center components [14,17]. Photosystem I is less sensitive to low temperatures than photosystem II and supports xanthophyll-mediated non-photochemical quenching, while maintaining active cyclic electron transport for ATP synthesis at low temperatures [8,11,18]. Chlororespiration and cyclic electron transport enable the dissipation of overproduced excitation energy via alternative electron acceptors, including the terminal oxidase in the plastid (PTOX), a bi-functional protein involved in carotenoid biosynthesis and oxidation of plastoquinol produced by over-reduced electron transport chain (ETC.) [19]–[23].

Potential excess NADPH produced as a result of linear photosynthetic electron transport in the almost complete absence of carbon fixation in the stroma can also be dissipated with or without altering ATP synthesis through the action of the alternate ETC. in mitochondria via operation of the plastidic malate/oxaloacetate shuttle [24]. The alternate mitochondrial ETC. also provides means of preventing ROS formation during mitochondrial aerobic respiration [25]. If ROS scavenging does not occur rapidly enough, under such conditions as extreme biotic or abiotic stresses including low temperatures, damage to mitochondrial and other cellular components and even programmed cell death may occur [26]–[30]. ROS production can also be prevented by lowering O_2_ levels and/or redirecting respiratory electron flux to other substrates. These alternate routes for electrons during respiration involve alternative oxidases, external and internal alternative NAD(P)H dehydrogenases, and uncoupling proteins, that are especially active upon imposition of abiotic stress that leads to ROS production [31]–[34]. These enzymes play a key role in plant acclimation and tolerance to low temperatures by preventing ROS formation when the traditional route involving complexes III and IV is inhibited [35]–[38]. These alternate routes bring about a lowering of ROS levels by maintaining active electron flow and preventing over-reduction of electron transport components, or redox imbalance [29,33,34,39,40].

Alternative oxidases compete for electrons with cytochrome c oxidase (complex III), using oxygen as a substrate. In addition to maintaining mitochondrial electron flow, they can also prevent ROS production directly by lowering the levels of oxygen, which can react with electrons to generate ROS, up to 60% during abiotic stresses [34,41,42]. Rotenone-insensitive alternative internal and external NAD(P)H dehydrogenases interfere with mitochondrial proton transport along with alternative oxidases [33,34,43]–[45]. These dehydrogenases bypass complex I, while alternative oxidases compete for the electrons with complex III, thus preventing a total of three protons from being transported from the matrix to the inter-membrane space by NADH dehydrogenase and cytochrome c reductase and oxidase [33,34]. Unlike alternative oxidases and dehydrogenases, uncoupling proteins diminish the electrochemical potential directly by transporting protons back to the matrix with the result that less ATP is synthesized. Uncoupling of the ETC. from oxidative phosphorylation stimulates fluxes through the ETC. and subsequently substrate oxidation during carbon and energy imbalance that occurs during a variety of stresses. As such, uncoupling proteins are also involved in controlling ROS levels and promoting tolerance of plants to stresses [31]–[34].

The transition from energy harvesting to dissipation of the photosynthetic apparatus in needles that takes place during winter hardening is a major adaptive response. However, freezing acclimation also involves a wide range of other cellular adaptations, which requires a substantial source of metabolic precursors and chemical energy that, given the circumstances, clearly cannot originate from linear photosynthetic electron transport and CO_2_ fixation. For example, detailed bioinformatic analyses of transcriptomic and metabolic remodeling during winter hardening in Sitka spruce (*Picea sitchensis*) revealed the presence of highly complex regulatory networks involving ethylene-mediated signaling, active trafficking of cell wall carbohydrates through the endomembrane system, and significant remodeling of cell walls [46]. Here we implement systems biology tools to further interrogate these datasets, which involve five time points sampled from late summer to early winter [47]. Specifically, the current paper focuses on adaptation of the photosynthetic apparatus to low temperature, with the focus on whole processes relevant to energy metabolism from the global perspective of systems biology rather than individual genes.

## Results

### Photosynthesis and respiration

**Figure 1:**
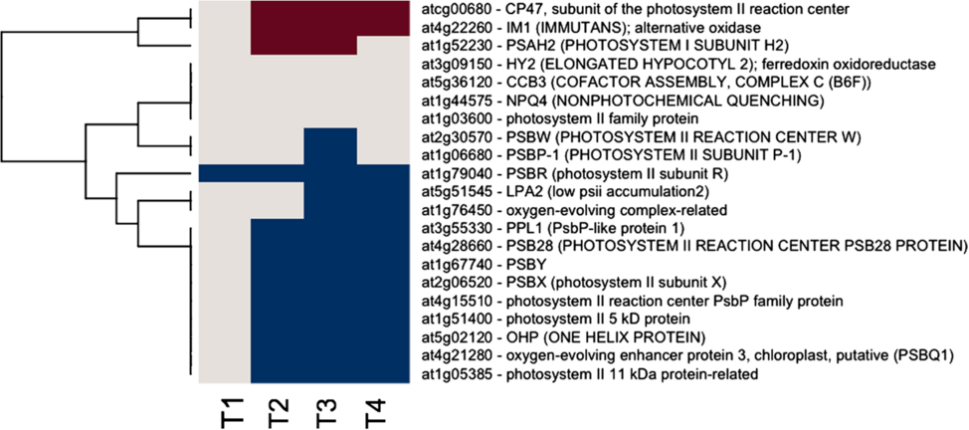
**Photosynthetic Gene Expression.** The gene expression levels across the rows are hierarchically clustered. Magenta cells represent significant positive gene expression changes, blue cells represent significant negative changes, and off-white cells represent no change or no statistically significant change. The level of significance used was at *P* ≤ 0.05.

**Figure 2:**
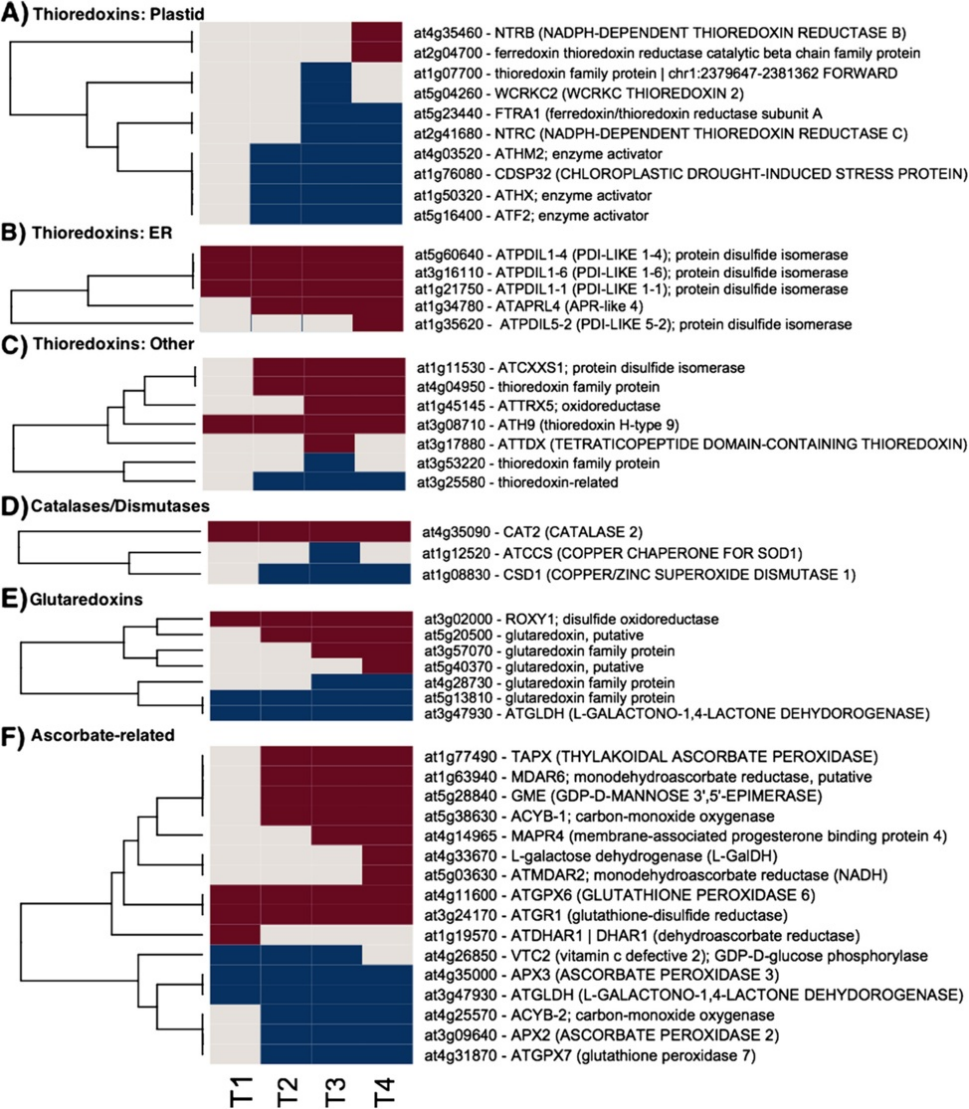
**Antioxidant Gene Expression.** The gene expression levels across the rows are hierarchically clustered. Magenta cells represent significant positive expression changes, blue cells represent significant negative changes, and off white cells represent no change or no statistically significant change. The level of significance used was at *P* ≤ 0.05. Genes and metabolites are grouped according to MapMan bin or MapMan bin combined with cellular location. The figure is divided into panels: **A**, thioredoxins transcripts associated with plastids, **B**, thioredoxins associated with the endoplasmic reticulum (ER), **C**, thioredoxins transcripts with unknown or locations other than plastids or endoplasmic reticulum, **D**, catalases and dismutases transcripts, **E**, glutaredoxin Transcripts, and **F**, ascorbate-related transcripts.

**Figure 3:**
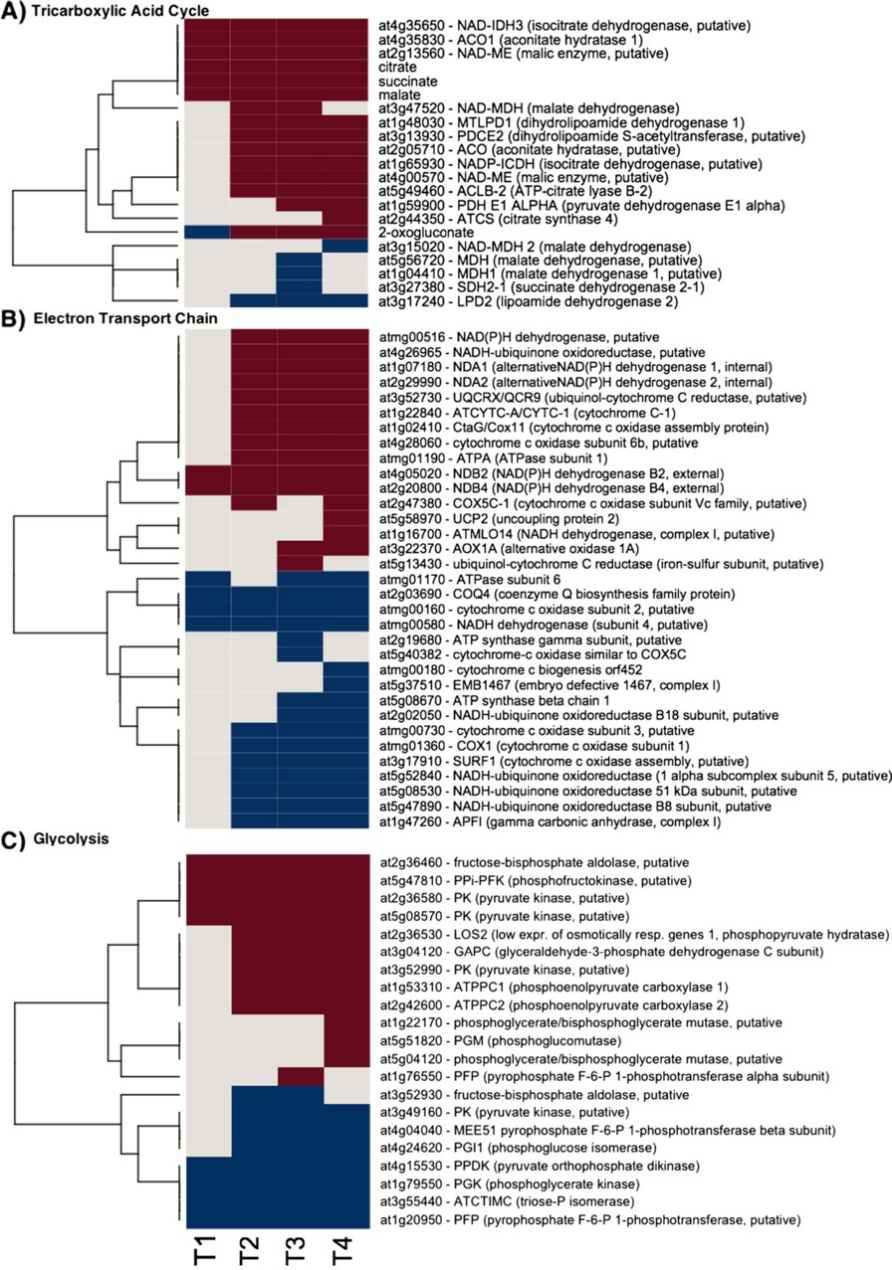
**Tricarboxylic Acid Cycle**, **ETC**., **and Glycolysis Gene Expression and Metabolite Changes.** The gene expression levels across the rows are hierarchically clustered. Magenta cells represent significant positive expression changes, blue cells represent significant negative changes, and off white cells represent no change or no statistically significant change. The level of significance used was at *P* ≤ 0.05. The figure is divided into panels: **A**, transcripts associated with the Tricarboxylic Acid Cycle, **B**, ETC. transcripts, and **C**, glycolysis related transcripts.

**Figure 4:**
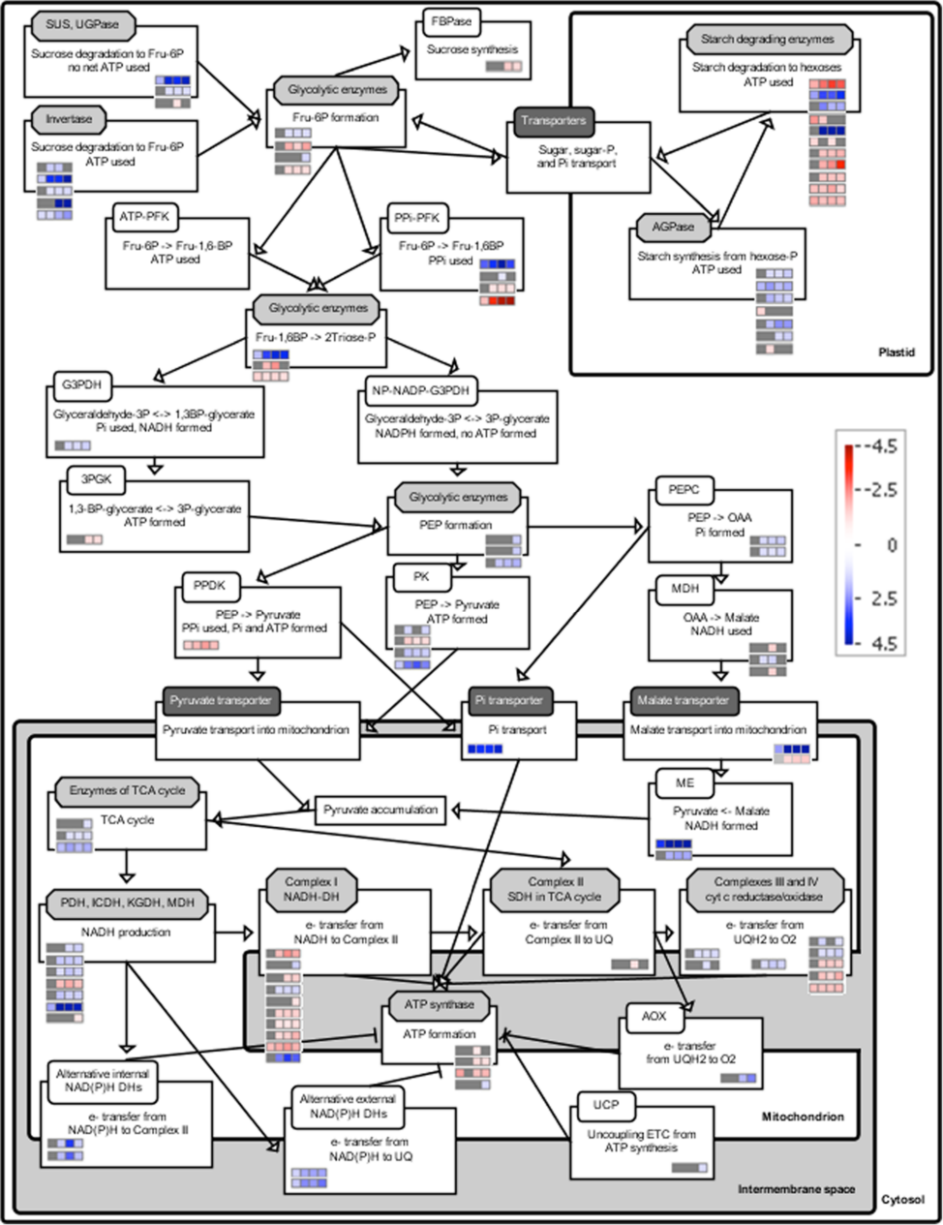
**SBGN Pathway of heterotrophic metabolism and changes in expression of metabolic genes in Sitka spruce needles during winter hardening.** Metabolic pathways were drawn using the BEACON tool to represent activities as boxed reactions with associated temporal changes in expression of relevant genes (rows in colored bins/small boxes) in TP1 through TP4 obtained from MapMan. Gray boxes indicate that the change in gene expression was not statistically significant, while blue and red indicate a statistically significant up- and down-regulation, respectively (*P* ≤ 0.05). Arrows connecting the activities indicate positive influence to generate a metabolic network, while “T” represents an inhibitory effect, e.g., components of the alternative ETC. in mitochondria interfere with adenosine triphosphate (ATP) synthesis. Each activity is associated with an enzyme (white rounded rectangle), enzyme complex (light gray octagon), or transporter (dark gray rounded rectangle). Abbreviations: 3PGK, 3-phosphoglycerate kinase; AGPase, ADP-glucose pyrophosphorylase; AOX, alternative oxidase; cyt, cytochrome; DH, dehydrogenase; FBPase, fructose-1,6-bisphosphatase; Fru, fructose; G3PDH, glyceraldehydes-3-phosphate DH; ICDH, isocitrate DH; KGDH, α-ketoglutarate DH; MDH, malate DH; ME, malic enzyme; NAD(P), oxidized nicotinamide adenine dinucleotide (phosphate); NAD(P)H, reduced nicotinamide adenine dinucleotide (phosphate); NP, non-phosphorylating; P, phosphate; PEP, phosphoenolpyruvate; PEPC, PEP carboxylase; PDH, pyruvate DH; PFK, phosphofructokinase; PK, pyruvate kinase; P_i_, inorganic phosphate; PP_i_, pyrophosphate; PPDK, pyruvate phosphate dikinase; SDH, succinate DH; SUS, sucrose synthase; TCA, tricarboxylic acid cycle; UCP, uncoupling protein; UGPase, UDP-glucose pyrophosphorylase; UQ/UQH_2_, oxidized/reduced ubiquinone.

The vast majority of responsive genes associated with chloroplast function were down-regulated after the time point 2 (TP2) and remained in that state for the duration of the time course (Figure 1 and Additional file 1: Table S1). Notable exceptions were *PTOX*/*IMMUTANS*, a nuclear gene encoding a plastid terminal oxidase, and *Psb*, a chloroplast gene encoding the CP47 subunit of the reaction center of Photosystem II (Figure 1), in addition to the plastid-targeted antioxidant gene products discussed below (Figure 2). In contrast, respiration-related genes were up-regulated (Figures 3 and 4). For example, *Atmg00516*, which encodes a subunit of mitochondrial NAD(P)H dehydrogenase (complex I), showed a consistent about 3-fold up-regulation throughout the time-course, and other components of the ETC. exhibited low, but consistent up-regulation, and clustered with the genes encoding alternative mitochondrial ETC. components. However, there were also corresponding sets of genes involved in respiration (both nucleus- and mitochondrion-encoded) that were down-regulated (Figures 3 and 4).

### Antioxidant function

Genes encoding proteins related to antioxidant activity in various organelles responded in diverse ways during the time course (Figure 2 and Additional file 2: Table S2). Massive increases in expression levels of peroxisomal catalase 2 were the most striking results in this overall category. Increases in the expression of genes encoding differentially targeted thioredoxin-related proteins were also observed, including three genes encoding protein disulfide isomerases (PDI gene family) targeted to the ER, and genes homologous to AtERO, which interacts with PDI in the ER. Genes encoding proteins that function in the ubiquitous ROS-scavenging ascorbate/glutathione pathway showed varying responses. Glutathione reductase 1, encoding a gene product targeted to the peroxisome and the cytosol, was up-regulated. Two monodehydroascorbate reductases, which generate ascorbate from monodehydroascorbate substrates, were up-regulated. One of these proteins is targeted to the chloroplast stroma. Two genes encoding ascorbate peroxidases were also up-regulated. After CAT2, thylakoid-associated L-ascorbate peroxidase was among the strongest up-regulated antoxidant genes. Re-reduction of ascorbate using reduced glutathione as a substrate was evident by the up-regulation of a dehydroascorbatereductase (subcellular location undefined), as well as an intrinsic trans-membrane cytochrome b561, and a membrane-associated heme binding protein that may be involved in intra-vesicular reduction of monodehydroascorbate. Within the glutaredoxin category, a spruce homolog of the disulfide oxidoreductase *ROXY1*, which has a regulatory role in angiosperm flower development, was highly up-regulated at the later time points. Notably up-regulated early in the time course were several genes involved in ascorbate biosynthesis, including an L-galactose dehydrogenase, which catalyzes the conversion of L-galactose to L-galactono-1,4-lactone, and a GDP-mannose 3,5-epimerase, which catalyzes an important step in the ascorbate biosynthetic pathway – the conversion of GDP-d-mannose to GDP-l-galactose.

The Automated Layout Pipeline for Inferred NEtworks (ALPINE) pipeline was used to visualize documented “guilt by association” relationships for the thylakoid-bound ascorbate peroxidase (tAPX) (Table 1). The “guilt by association” relationships are imported from GeneMania and drawn from a large dataset containing functional associations to tAPX. These functional associations include protein and genetic interactions, pathways, co-expression, co-localization and protein domain similarity. The ALPINE visualization integrates the functional associations of genes, Gene Ontology (GO) annotations, and subcellular location data into a single unified view. This view is filtered by user supplied gene expression data to return only gene associations obtained from the input. This ability to exclusively analyze a particular dataset and return only the gene associations of interest and significance is unique to ALPINE and to our knowledge, there are no bioinformatics tools available with the ALPINE’s useful and unique combination of functionalities and capabilities.This strategy revealed clues concerning antioxidant-related events in winter hardening that are not apparent using other singular strategies. Of the 28 genes that were found to be associated with tAPX, all but five were down-regulated, and all but one gene encoded a plastid-targeted protein. The plastid-targeted down-regulated genes fell into several categories, for example, photosystem II, chloroplast-based protein synthesis, protein folding, and RNA regulation of transcription. In contrast, a gene encoding a member of the rhodanase family was up-regulated, as was a plastid-targeted, NADP-linked oxidoreductase. Taken together, tAPX may be associated with the regulatory mechanism that brings about the virtual cessation of many chloroplast activities that occur during winter hardening.

**Table 1:** **Gene association network generated by querying thylakoid ascorbate peroxidase** (**tAPX**, **AT1G77490**) **using the ALPINE tool**

AT1G77490	thylakoidal ascorbate	Plastid	PlastidRedox.Ascorbate and Glutathione.Ascorbate	3.41	1.51E-04
AT4G39970	Haloacid dehalogenase‒like hydrolase (HAD) superfamily	Plastid	Not Assigned. No Ontology	2.53	1.15E-03
AT5G51820	phosphoglucomutase	Plastid	Glycolysis.Plastid Branch.Phosphoglucomutase	1.5	4.98E-02
AT4G27700	Rhodanese/Cell cycle control phosphatase superfamily	Plastid	Misc.Rhodanese	1.48	8.16E-03
AT1G04420	NAD(P)‒linked oxidoreductase superfamily protein	Plastid	Minor CHO Metabolism.Others	1.47	4.78E-04
AT5G27290	AT5G27290unknown protein	Plastid	Not Assigned.Unknown	1.36	4.71E-02
AT5G65220	AT5G65220Ribosomal L29 family protein	Plastid	Protein.Synthesis.Ribosomal Protein.Prokaryotic.	−1.32	4.09E-02
AT4G21280	AT4G21280photosystem II subunit QA	Plastid	PS.Lightreaction.Photosystem II.PSII Polypeptide Subunits	−1.33	1.45E-02
AT1G32550	2Fe‒2S ferredoxin‒like superfamily protein	N/A	Misc.Other Ferredoxins and Rieske Domain	−1.33	4.95E-02
AT1G76450	Photosystem II reaction center PsbP family protein	Plastid	PS.Lightreaction.Photosystem II.PSII Polypeptide Subunits	−1.37	1.34E-02
AT2G43560	FKBP‒like peptidyl‒prolyl cis‒trans isomerase family protein	Plastid	Protein.Folding	−1.37	1.54E-02
AT3G52150	RNA‒binding (RRM/RBD/RNP motifs) family protein	Plastid	RNA.Regulation of Transcription.Unclassified	−1.41	2.59E-02
AT3G18890	NAD(P)‒binding Rossmann‒fold superfamily protein	Plastid	Signalling.Light	−1.45	2.02E-02
AT3G51510	unknown protein	Plastid	Not Assigned.Unknown	−1.46	1.54E-02
AT1G48350	Ribosomal L18p/L5e family protein	Plastid	Protein.Synthesis.Ribosomal Protein.Prokaryotic.Chloroplast	−1.46	1.54E-02
AT1G43670	Inositol monophosphatase family protein	Membrane	Metabolism.Synthesis.Sucrose.F	−1.51	3.97E-02
AT1G75690	DnaJ/Hsp40 cysteine‒rich domain superfamily protein	Plastid	Protein.Folding	−1.6	4.19E-02
AT5G14910	Heavy metal transport/detoxification	Plastid	Not Assigned.No Ontology	−1.62	3.44E-02
AT1G21350	Thioredoxin super family protein	Plastid	Not Assigned.Unknown	−1.63	1.33E-02
AT5G13510	Ribosomal protein L10 family protein	Plastid	Protein.Synthesis.Ribosomal Protein.Prokaryotic.	−1.69	3.43E-05
AT3G48420	Haloacid dehalogenase‒like hydrolase (HAD) superfamily	Plastid	Not Assigned.Unknown	−1.7	2.50E-02
AT1G16080	unknown protein	Plastid	Not Assigned.Unknown	−1.73	4.36E-03
AT4G09650	ATP SYNTHASE DELTA‒SUBUNIT; hydrogen ion	Plastid	Not Assigned.Unknown	−1.9	3.44E-04
AT3G54210	Ribosomal protein L17 family protein	Plastid	Protein.Synthesis.Ribosomal Protein.Prokaryotic.Chloroplast.	−1.92	4.44E-04
AT3G61870	unknown protein	Plastid	Not Assigned.Unknown	−2.06	2.71E-03
AT5G52970	thylakoid lumen 15.0 kDa protein	Plastid	Not Assigned.No Ontology	−2.06	4.12E-04
AT4G01310	Ribosomal L5P family protein	Plastid	Protein.Synthesis.Ribosomal Protein.Prokaryotic.	−2.42	8.36E-04
AT5G42070	unknown protein	Plastid	Not Assigned.Unknown	−3.01	2.08E-05

All associations were found to be co-expression associations, and all 28 genes in the network demonstrate a significant (*q* <0.05) expression value in T5 of the dataset.

### Alternative ETC

Components of the alternative electron transport are known to interfere with mitochondrial ATP production. Two external alternative NAD(P)H dehydrogenases showed a consistent up-regulation in response to freezing temperatures across the time series, while two internal ones from TP2 to TP4 (Figures 3 and 4). Alternative oxidase A1 gene (*At3g22370* in Arabidopsis) was up-regulated at TP3 and 4, while an ortholog of uncoupling protein 2 (*At5g58970*) at TP4 only (Figures 3 and 4).

### Carbon and reductant sources for respiration

**Figure 5:**
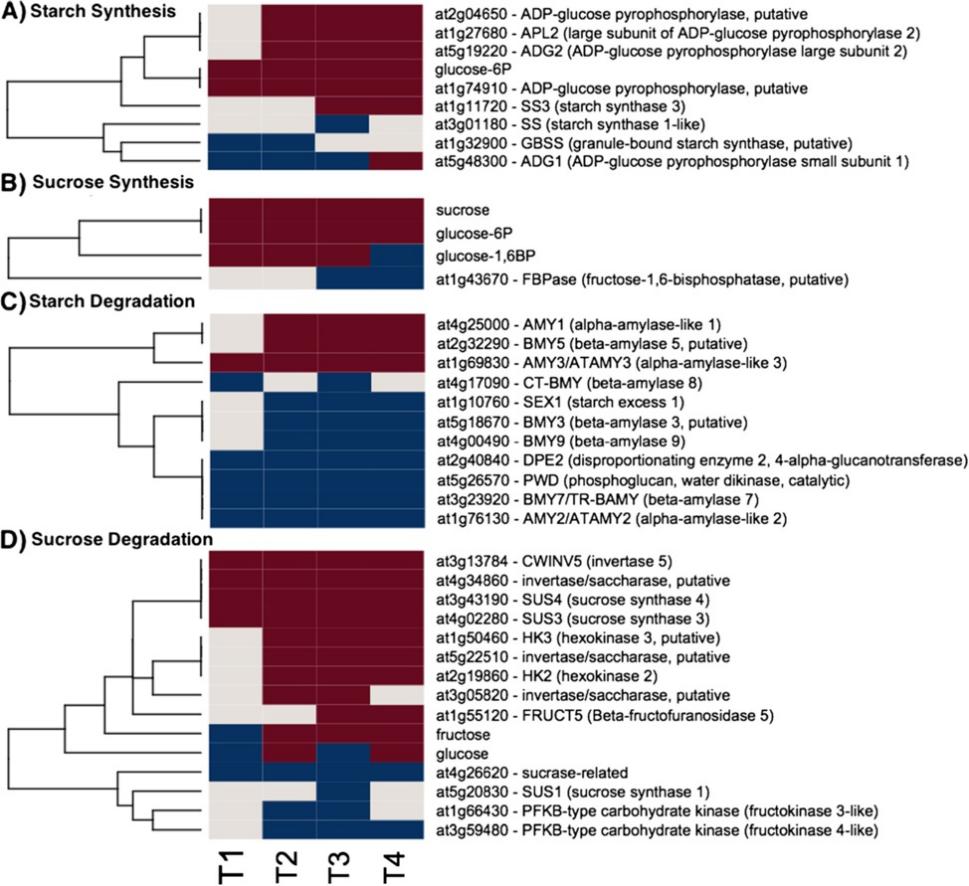
**Starch and Sucrose Metabolism Gene Expression and Metabolite Changes**. The gene expression levels across the rows are hierarchically clustered. Magenta cells represent significant positive expression changes, blue cells represent significant negative changes, and off white cells represent no change or no statistically significant change. The level of significance used was at *P* ≤ 0.05. The figure is divided into panels: **A**, transcripts associated with starch synthesis, **B**, sucrose synthesis, **C**, starch degradation, and **D**, sucrose degradation.

To determine the source of reductant for mitochondrial electron transport, we assessed changes in gene expression of various genes involved in central carbon metabolic pathways that could provide these substrates (Additional file 3: Table S3). The only potentially active pathways involved sugars. Transcript levels of four invertase genes, which are involved in starch degradation, increased, including a cell wall invertase, whose expression level increased through TP4 (Figures 4 and 5). A homolog of *At4g34860*, a cytosolic invertase, showed large increases through TP4. The expression of homologs to members of the sucrose synthase (SUS) gene family showed a somewhat analogous pattern with SUS4 exhibiting a substantial increase through TP4. SUS3 also showed an increase, albeit a more modest one, which peaked between TP3 and TP4. In contrast, SUS1 showed a decrease in TP3 and no significant changes at other TPs. However, sucrose levels remained constant and above baseline levels during freezing acclimation. More generally, genes encoding starch biosynthesis enzymes were moderately up-regulated, while those associated with degradation were down-regulated (Figures 4 and 5). ADP glucose pyrophosphorylase (AGPase) involved in starch biosynthesis is a heteromer of two large and two small subunits. APL1 and 2, two of the three genes encoding the large AGPase subunits responded and showed increases in the expression from TP2 to TP4. A gene encoding the small subunit of AGPase (APS1) showed a decrease in expression at TP1, 2 and 3 and an increase in TP4. One gene encoding starch synthase showed an initial decrease in TP1 and 2, while the other responsive starch synthase gene was down-regulated at TP3 (Figures 4 and 5).

Most genes encoding glycolytic enzymes did not have considerably altered levels of relevant transcripts in response to freezing (Figures 3 and 4). However, some glycolytic genes showed a more substantial up- or down-regulation, including the pyrophosphate-dependent phosphofructokinase 2 (*At5g47810*) that showed a maximal 5.2-fold increase and a constant up-regulation. The same step in glycolysis can also be catalyzed by another pyrophosphate-dependent enzyme, fructose-6-P 1-phosphotransferase and the corresponding α - subunit (*At1g20950*) was correspondingly down-regulated (a maximal 5.2-fold decrease). Fructose-1,6-bisP aldolase (*At2g36460*) transcript levels increased consistently (~4-fold) in response at all TPs. In Arabidopsis, *At5g08570* encodes a protein similar to cytosolic pyruvate kinase, and the corresponding gene in Sitka spruce showed a 3-fold increase in transcript levels. Transcript levels of gluconeogenetic pyruvate pyrophosphate dikinase (*At4g15530*) decreased at TP1 and remained low throughout the time course. Phospho*enol*pyruvatecarboxykinase (encoded by *At4g37870* in Arabidopsis) transcript gradually decreased 4.4 fold through TP4.

Dihydrolipoamide dehydrogenase 1 (LPD1, *At1g48030*), the E3 subunit 1 of pyruvate dehydrogenase, was moderately (up to 1.8-fold), but significantly up-regulated from TP2 to TP4 (Figures 3 and 4). In contrast, LPD2 (*At3g17240*), the E3 subunit 2 of pyruvate dehydrogenase, was down-regulated about 1.8-fold between TP2 and TP4. The steady-state transcript levels for other subunits of pyruvate dehydrogenase remained unchanged during freezing. This was true for most of the transcripts encoding the remaining enzymes of the TCA cycle, though they tended to be slightly above the baseline levels (typically less than a 1.5-fold change) throughout the time course. A putative cytosolic NADP-dependent isocitrate dehydrogenase (*At1g65930*) showed the same pattern and degree of increases in gene expression as LPD1. A putative regulatory subunit of the mitochondrial NAD-dependent isocitrate dehydrogenase III (*At4g35650*) increased 2-fold in TP1 and showed a stable 4.4-fold increase in transcript levels in TP2 through TP4. Malic enzyme gene (*At4g00570*) showed only a moderate 2.2-fold up-regulation in response to freezing, but the consistent 9- to 11-fold induction of the *At2g13560*-encoded NAD-dependent malic enzyme transcript levels in TP2, 3, and 4 was quite striking. Citrate and succinate levels were moderately increased in a stable manner, while malate levels gradually increased up to 2.9 fold during freezing (Figure 4).

## Discussion and conclusions

Temperate and boreal conifers must survive harsh winters while retaining their photosynthetic tissue, and have evolved intricate mechanisms to maintain a certain level of active metabolism, signaling, and protective processes at low temperatures in their needles. The comprehensiveness of our data afforded a global view of winter hardening as it is reflected in numerous processes. The use of bioinformatics tools enabled the visualization of these changes in a cellular context. The results point to numerous photoprotective processes that are active during winter, serve to confirm previous reports, and provide new insights into the activity of genes associated with chloroplast function, energy metabolism, antioxidant processes, and possible regulatory mechanisms.

Genes related to energy quenching mechanisms including a spruce PsbS homolog and plastid terminal oxidase PTOX increased their expression, which is in an agreement with previous reports [10]. PsbS activity is associated with changes in photosystem II that occur as part of the photoprotective mechanisms that are set in place as the temperature drops, while PTOX represents an important component of the dissipation of excess light energy in photosynthesis as an alternative electron acceptor and O_2_-utilizing enzyme helping to prevent ROS formation in hardening conifer needles [10]. In *Ranunculus glacialis*, an alpine plant growing in high altitudes at high light and low temperatures, PTOX protein levels correlated with the use of alternative ETC. and enabled high fluxes through photosynthetic ETC. despite the excess of light energy. PTOX was implicated to serve as a safety valve for the over-reduction of photosystems during high light and low temperatures in alpine plants [48]. PTOX has also been implicated in increasing the flux through the linear ETC. and subsequent increased tolerance of photosystems to high light in cold-hardened *Arabidopsis thaliana*[49]. This result suggests that the energy dissipation mechanisms set in motion within the chloroplast during winter hardening were effective. One manifestation of this may have been a relatively low level of ROS generation as the temperature dropped. However, redox effects on cellular responses to the environment are not confined to a simple containment of ROS. Much data point to regulatory roles for specific antioxidant proteins in plant cells under stress. Several of these antioxidant, regulatory genes were up-regulated over the winter hardening time course, including catalase CAT2, glutaredoxin ROXY1, and glutathione reductase GR1. Catalases regulate the levels of hydrogen peroxide, which is an important signaling molecule in plant stress responses [50,51]. *ROXY1*encodes a glutaredoxin with a documented regulatory role in development in Arabidopsis [52]–[54], while GR1exerts a regulatory effect on responses to cold stress, an effect that cannot be replaced by GR2, or thioredoxins [55].

The ROS scavenging pathway may have been operational in the chloroplast as winter hardening proceeded, since other components, such as mondehydroascorbate reductase, were also up-regulated (Table 1). However, it is also possible that tAPX was acting in a different/additional capacity to protect the photosynthetic machinery. Duan et al. (2012) have shown that tAPX plays a specific role in alleviating the photosystem I and II-associated photoinhibition that occurs with chilling stress in Arabidopsis [56], and spruce tAPX may play an analogous role in the chloroplasts of hardening needles. Interestingly, Maruta et al. (2010) provide evidence for a specific regulatory role for tAPX in chloroplast responses to stress, resonant with the role shown for GR1 [57]. In the case of tAPX and the winter hardening data, the guilt by association analysis revealed an association of tAPX exclusively with plastid-targeted gene products, in which the great majority of those responsive genes were down-regulated. It is possible that tAPX plays an important role in the adaptation mechanism that results in the onset of chloroplast dormancy in conifer needles. Taken together, these results suggest specific regulatory roles for *tAPX*, *ROXY1*, and *GR1* in the adaptation mechanism underlying winter hardening.

Freezing temperatures impose severe stress on the mitochondrial ETC. A set of genes encoding components of the respiratory ETC. were strongly or mildly up-regulated, suggesting that the mitochondrial ETC. is active. Mitochondrial aerobic respiration is a source of ROS formed through the reduction of molecular oxygen by electrons coming from complexes I and III of the ETC. [25]. The ROS production can be prevented by redirecting respiratory electron flux to other substrates by involving the major players of the alternate ETC. including alternative oxidases, external and internal alternative NAD(P)H dehydrogenases, and uncoupling proteins [31]–[34]. Genes encoding all these components of the alternate ETC. were up-regulated during the time course, suggesting that these alternate pathways are active, along with the conventional ETC., during freezing acclimation. Up-regulation of genes encoding the components of the alternate ETC. is typical during cold acclimation in plants in general and is accompanied with metabolic changes. Tobacco plants subjected to cold showed an increase in alternative oxidase levels and sugar content compared to the transgenic plants with suppressed expression of this enzyme [58]. These transgenic plants also showed an increased lipid peroxidation at normal, but not at low, temperatures. Alternative oxidases are regulated at the transcriptional level by abscisic acid insensitive 4 (ABI4) transcription factor and are activated by pyruvate [59]–[61]. Accumulation of pyruvate as the final product of glycolysis indicates an imbalance between glycolytic fluxes and the mitochondrial ETC. Pyruvate then activates alternative oxidases to stimulate flux through the mitochondrial ETC. to keep pace with glycolysis [34,62,63]. Suppressing the external NAD(P)H dehydrogenase NDB4 resulted in increased levels of NDB2 and alternative oxidase 1A in tobacco, which lead to increased salt tolerance and improved growth [45].

### Glycolysis and the TCA cycle as sources of carbon, ATP, and reductant

Avoiding ROS production comes at a price, as every alternate route leads to a decreased ATP production by mitochondrial ATP synthase. Both normal and alternate ETCs were active based on our data, which brings a question of the potential source(s) of reductant for these ETCs and source(s) of ATP that would supplement ATP lost due to the potentially high activities of the alternate routes for electrons. Most glycolytic genes did not show major steady-state transcript differences during freezing acclimation, though persistent up-regulation was observed, suggesting that glycolysis was active. In plants, glycolysis is regulated at the transcriptional and allosteric levels and in a complex way due to metabolic flexibility [33,34,64]. In situations when ATP cannot be easily produced in heterotrophic systems (e.g., during hypoxia, chilling, or phosphate starvation), pyrophosphate- instead of ATP-utilizing enzyme forms are used to drive glycolysis [33,34,65,66]. One of the genes encoding phosphofructokinase was up-regulated up to 5.2-fold during winter hardening. Up-regulation of a pyrophosphate-dependent phosphofructokinase isoform suggests the possibility of conservation of ATP during freezing acclimation, similar to phosphate starvation stress. To maximize ATP production, alternate glycolytic steps involving substrate-level phosphorylation are preferentially utilized. The conversion of glyceraldehyde-3P to 3-phosphoglycerate can be done in two ways by: (i) non-phosphorylating glyceraldehyde-3P dehydrogenase in a single step to only produce NADPH and (ii) phosphoglycerate kinase and phosphorylating glyceraldehyde-3P dehydrogenase in two steps involving NADH and ATP production [34]. The phosphorylating glyceraldehyde-3P dehydrogenase was moderately, but consistently up-regulated, while the kinase showed a consistent, less than 1.5-fold down-regulation, suggesting that the two-step reaction producing both the reductant and ATP could be active. ATP production by substrate-level phosphorylation appears to be active in general – three transcripts coding for pyruvate kinase showed varying, but consistent up-regulation. Pyruvate kinase catalyzes a key step regulating glycolysis and pyruvate is a known allosteric regulator of a number of glycolytic enzymes including pyruvate kinase, activity of which is induced during hypoxia when glycolysis is the major source of ATP [33,34]. Based on these observations, it appears that glycolytic routes conserving or producing ATP are preferentially used. This is in an agreement with the global activation of alternative ETC. routes. As such, glycolysis could be a source of ATP supplementing lower ATP levels obtained from respiration and could help with high-energy cofactor balancing.

In conventional glycolysis, pyruvate kinase converts phospho*enol*pyruvate to pyruvate and ATP is made. However, mitochondria could oxidize substrates other than pyruvate, such as malate [33], during winter hardening. In this scenario, the pyruvate kinase step is bypassed by three consecutive steps with no net NADH and ATP production or consumption (Figure 4). However, this bypass allows NADH from the cytosol to be shuttled inside mitochondria where it can be used by the conventional or alternative ETCs. First, cytosolic phospho*enol*pyruvate is carboxylated to oxaloacetate by phospho*enol*pyruvate carboxylase, a highly regulated enzyme whose activity increases during phosphate starvation [67]–[70]. Second, oxaloacetate is reduced to malate by cytosolic NADH-utilizing malate dehydrogenase, and malate is transported to mitochondria. The dicarboxylate/tricarboxylate transporter DTC encoded by the Arabidopsis *At5g19760* gene ortholog that is capable of transporting malate to mitochondria [71] showed a consistent 5-fold up-regulation. Third, malate either enters the TCA cycle or is decarboxylated to pyruvate by NAD-dependent malic enzyme in mitochondria. Most genes encoding the enzymes of the TCA cycle showed a moderate up-regulation or no significant changes in their steady-state transcript levels, suggesting that the TCA cycle is operational. For example, a putative regulatory subunit of the mitochondrial NAD-dependent isocitrate dehydrogenase III (*At4g35650*) [72,73] showed a stable 4.4-fold increase in transcript levels in TP2 through TP4. Malic enzyme has been implicated in generating NADH and pyruvate needed for lipid synthesis in heterotrophic and photoheterotrophic systems such as plant seeds [74]–[81].

The physiological significance of this bypass in most *in vivo* situations is not well understood [33]. However, existing metabolomic and transcriptomic data [82] suggest that the bypass is operational (along with the conventional pyruvate kinase step) during freezing acclimation. Malate levels increased significantly at TP1 and reached the levels that were nearly 3-fold higher than baseline later in the time course. Two genes encoding phospho*enol*pyruvate carboxylase and one gene encoding malic enzyme were moderately up-regulated in response to freezing acclimation, while cytosolic malate dehydrogenase transcript levels were not significantly different in all TPs. However, the Sitka spruce homolog of the Arabidopsis *At2g13560*-encoded NAD-dependent malic enzyme 1, which is allosterically activated by fumarate [83], showed a 9- to 11-fold up-regulation in its transcript levels during freezing acclimation and could drive significant fluxes through this bypass. Low temperature stress was also shown to induce the expression of a gene encoding malic enzyme in maize [24]. To drive efficient conventional and alternative ETCs during winter hardening, this bypass could: (i) shuttle NADH from cytosol to mitochondria, (ii) bring additional carbon from glycolysis to the TCA cycle to stimulate alternative and conventional mitochondrial ETCs, and (iii) generate phosphate for ATP synthesis.

### Potential sources of hexoses for glycolysis

The reaction catalyzed by invertase requires 2 ATP molecules to generate fructose-6P, while the net NTP consumption for the sucrose synthase pathway is zero and the latter is up-regulated during hypoxia to save ATP [84]. Both invertases and sucrose synthases showed significant increases in the corresponding transcript levels in Sitka spruce needles during freezing acclimation. However, steady-state sucrose levels did not change significantly. So, if there were an increase in sucrose degradation to provide glucose and fructose for glycolysis, there would be a corresponding increase in sucrose supply to maintain unchanging sucrose levels. Because photosynthetic carbon fixation is close to inactive, one can hypothesize that sucrose would have to be transported from organs that have a great potential to store it or made from other, more condensed form of carbohydrate such as starch that can easily be converted to sucrose.

Starch can accumulate transiently in photosynthetically active cells or be stored for a prolonged period of time in tubers and seeds [85]–[89]. This makes starch an excellent candidate as a storage compound that could be mobilized and used as a source of hexoses during winter hardening. However, the majority of genes associated with starch degradation were down-regulated, while those involved in its biosynthesis were up-regulated, which would make starch present in needles a seemingly unlikely storage form of carbon. Regulation of starch metabolism is complex, as it involves several levels of regulation, including transcriptional, protein phosphorylation, allosteric, and redox regulation, to contribute to the balance between photosynthate production and demand in source and sink tissues [87,90]–[92] and as such, transcript levels alone cannot provide enough information regarding the direction of starch metabolism during winter hardening. Changes in starch levels were not measured during freezing acclimation in Sitka spruce needles [82], but Fisher &Holl [93] showed that starch content progressively decreased in Scots pine needles during the winter, suggesting that this polysaccharide could possibly serve as a storage compound for the winter hardening processes also in Sitka spruce needles despite the up-regulation in starch synthesis genes. However, an increase in starch synthesis was also observed in Arabidopsis leaves exposed to prolonged moderate osmotic stress [94] that lead to similar global gene expression responses involving cell wall remodeling, vesicular transport, and hormonal signaling as in winter hardening [46], but the reason for these increases in starch synthesis remains to be elucidated.

Addressing the question of the storage form of carbon in needles during winter hardening is quite complicated. Starch is a potential candidate, but conifers also accumulate di and triacylglycerols as storage compounds in the needles [93]. Unfortunately these compounds were not measured in Sitka spruce needles during winter hardening [82], but both types of lipids showed a similar trend as their levels gradually increased during fall and winter in Scots pine needles, followed by a rapid decrease in spring prior to bud break, suggesting that lipid degradation provides additional carbon and energy for new buds before photosynthesis is fully operational [93]. It is unclear whether these lipids can also be used as a source of energy during winter hardening. Starch levels decreasing and lipid levels increasing in needles during the winter is consistent with the hypothesis that starch rather than lipids are substrates for respiration and active cell-wall remodeling during winter. In addition, degradation of oligo and polysaccharides is more energy effective for producing hexoses for substantial cell-wall remodeling than potential gluconeogenesis (down-regulated during winter hardening) from lipids. A possibility also exists that soluble and insoluble sugars are stored in other parts of the trees and then transported in the form of sucrose to needles to help maintaining active metabolism during prolonged freezing. All these compounds including di and triacylglycerols are present in the sapwood of Scots pine [93], but detailed source-sink relationship studies are needed to confirm the possibility of sucrose transport to needles in winter.

## Methods

### Plant material, microarray analysis, and metabolite profiling

Plants and gene expression data for this study are described in [47]. Sitka spruce plants originating from Prince Rupert, British Columbia, Canada, were grown in an outdoor, raised-bed garden at Vancouver, British Columbia, Canada. In the fourth growth season (2004), needle tissue from plants were sampled for RNA extraction on August 30, October 18, November 22, December 1, and December 13. Expression changes associated with these time points are referred to as TP1 for the ratio of October 18:August 30, TP2 for the ratio of November 22:August 30, and so on. Microarray analysis was performed using a 21.8K spruce cDNA microarray. The slides were scanned and quantified using ImaGene software (BioDiscovery, Inc., El Segundo, CA), and subsequently normalized using variance stabilizing normalization. Fold-changes and probability values for each cDNA clone were calculated using a linear model in which the normalized intensity values in the Cy3 and Cy5 channels were adjusted for dye and array effects [47]. In addition to transcriptome analysis, data on soluble metabolites quantified by gas chromatography–mass spectrometry (GC-MS) were taken from [82] for the same samples [47]. All statistical analyses were carried out using the R statistical package (http://www.r-project.org).

### Metabolic mapman

The MapMan tool enables the classification, statistical analysis, and visualization of transcripts and metabolites into hierarchical categories (known as bins) without the redundancies encountered when using other ontologies [95]. A modification to MapMan that provides a view of multiple instances of a bin simultaneously, e.g., to enable visualization of time courses, was implemented.

### BEACON

Beacon is a software and database system designed for the construction, editing, annotation, and storage of signal transduction pathways. For the representation of pathway entities, Beacon implements a graphical language known as the Activity Flow (AF) language, which is part of a set of standardized graphical languages defined in the Systems Biology Graphical Notation project [96]. The AF language portrays pathways at the level of biological activities, phenotypes, and perturbations, with a particular focus on the influences that these entities have on one another in a given pathway. The language is of particular use in the representation of signal transduction pathways due to the flexibility inherent in these viewpoints of biological entities. The Beacon Pathway Editor, specifically, refers to the graphical user interface component for the construction, editing, and annotation of signal transduction pathways using the AF graphical language. A beta version of the Beacon Pathway Editor has been released to a select group of users and is expected to be available to the public in the fall of this year. Once completed, the Beacon Pathway Editor will be extended to allow for the simulation of user-constructed pathways, enabling the possibility for “what if” analyses by manipulating the states of biological activities and perturbations in a given pathway. The database used to store Beacon pathways is also in development, with one eventual goal being the creation of an inference engine to infer new connections among pathway entities based on the information contained in all stored pathways.

### ALPINE

The Automated Layout Pipeline for Inferred NEtworks (ALPINE) tool is an in-house project that is currently in the early stages of development. The purpose of the tool is to make the generation of inferred gene association networks, the filtering of those networks to include only those genes that are significant in a user-provided gene expression data set, and the layout of those networks based on subcellular localization annotations an entirely automated process. At present, the tool is implemented as a plugin for the Cytoscape [97] visualization environment to act as an intermediary between the existing GeneMANIA and Mosaic Cytoscape plugins. The GeneMANIA plugin allows users to enter a set of query genes and to generate an inferred gene association network for a variety of association types (physical interactions, genetic interactions, co-expression associations, and more) [98]. The Mosaic plugin allows users to partition, color, and layout existing networks based on GO annotations and other network attributes [99]. The ALPINE plugin provides a graphical user interface that allows the user to set the required parameters for network inference via GeneMANIA and a network layout via Mosaic. Additionally, the user interface allows the user to load a gene expression data set, and to filter the generated network to include only those genes with significant expression values in a minimum number of time points specified by the user. The ultimate result is that, once the user has entered the required parameters, the tasks of network inference, filtering, and layout are handled automatically by the ALPINE tool.

For the networks generated using ALPINE in the current paper, we required a greater specificity in the subcellular localizations annotated to each gene than those provided by the Mosaic portion of ALPINE. In order to achieve a greater specificity, the resulting Cytoscape networks were uploaded to the Arabidopsis Interactions Viewer [100], one of many available tools included in the Bio-Array Resource for Plant Biology collection, where more specific subcellular localizations, based on data from the Arabidopsis Subcellular Database, were annotated to each gene and a layout was performed to group genes based on subcellular localizations. Optional use of the Arabidopsis Interactions Viewer for subcellular localization annotations is currently not included in the ALPINE tool; however, it is possible that the tool may include such an option before it is released for public use.

## Abbreviations

ABI: Abscisic acid insensitive; ADP: Adenosine diphosphate; AF: Activity flow; AGPase: ADP glucose pyrophosphorylase; ALPINE: Automated layout pipeline for inferred networks; ATP: Adenosine triphosphate; cDNA: Complementary deoxyribonucleic acid; DTC: Dicarboxylate/tricarboxylate transporter; ER: Endoplasmic reticulum; ETC: Electron transport chain; GC-MS: Gas chromatography–mass spectrometry; GDP: Guanosine diphosphate; GR: Glutathione reductase; GO: Gene ontology; LPD: Dihydrolipoamide dehydrogenase; NAD(P)H: Reduced nicotinamide adenine dinucleotide (phosphate); NTP: Nucleotide triphosphate; PDI: Protein disulfide isomerases; PTOX: Plastidic terminal oxidase; RNA: Ribonucleic acid; ROS: Reactive oxygen species; SUS: Sucrose synthase; tAPX: Thylakoid-bound ascorbate peroxidase; TCA: Citric acid cycle; TP: Time point.

## Competing interests

The authors declare that they have no competing interests.

## Authors’ contributions

EC, RG, JAH, and LSH developed the concept of this computational study. EC, RG, and JAH interpreted and discussed biological findings and concepts of the computationally derived results. EC prepared Figure 4. CK, HS, and EM performed all computational analyses under the supervision of RG and LSH and prepared all other figures and tables. All authors reviewed and approved the final manuscript.

## Supplementary Material

Additional file 1: Table S1 TCA and Electron Transport. Raw data file showing MapMan bin, Arabidopsis thaliana homolog ID, annotations, time point expression, and location. Click here for file

Additional file 2: Table S2 Antioxidants. Raw data file showing MapMan bin, Arabidopsis thaliana homolog ID, annotations, time point expression, and location. Click here for file

Additional file 3: Table S3 Starch and sucrose metabolism. Raw data file showing MapMan bin, Arabidopsis thaliana homolog ID, annotations, time point expression, and location. Click here for file
